# Applying the moving epidemic method to determine influenza epidemic and intensity thresholds using influenza‐like illness surveillance data 2009‐2018 in Tunisia

**DOI:** 10.1111/irv.12748

**Published:** 2020-05-10

**Authors:** Hind Bouguerra, Elyes Boutouria, Mokhtar Zorraga, Amal Cherif, Rihab Yazidi, Naima Abdeddaiem, Latifa Maazaoui, Awatef ElMoussi, Salma Abid, Slim Amine, Leila Bouabid, Souha Bougatef, Mohamed Kouni Chahed, Afif Ben Salah, Jihene Bettaieb, Nissaf Bouafif Ben Alaya

**Affiliations:** ^1^ National Observatory of New and Emerging Diseases Ministry of Health Tunis Tunisia; ^2^ Direction of Primary Health Care Tunis Tunisia; ^3^ Pasteur Institute of Tunis Tunis Tunisia; ^4^ Microbiology Laboratory Virology Unit Charles Nicolle Hospital Tunis Tunisia; ^5^ Faculté de Médecine de Tunis Université de Tunis El Manar Tunis Tunisia; ^6^ Faculté de Médecine de Tunis LR01ES04 Epidémiologie et Prévention des Maladies Cardiovasculaires en Tunisie Université de Tunis El Manar Tunis Tunisia

**Keywords:** epidemic threshold, ILI surveillance, influenza, moving epidemic method

## Abstract

**Background:**

Defining the start and assessing the intensity of influenza seasons are essential to ensure timely preventive and control measures and to contribute to the pandemic preparedness. The present study aimed to determine the epidemic and intensity thresholds of influenza season in Tunisia using the moving epidemic method.

**Methods:**

We applied the moving epidemic method (MEM) using the R Language implementation (package “mem”). We have calculated the epidemic and the different intensity thresholds from historical data of the past nine influenza seasons (2009‐2010 to 2017‐2018) and assessed the impact of the 2009‐2010 pandemic year. Data used were the weekly influenza‐like illness (ILI) proportions compared with all outpatient acute consultations. The goodness of the model was assessed using a cross validation procedure.

**Results:**

The average duration of influenza epidemic during a typical season was 20 weeks and ranged from 11 weeks (2009‐2010 season) to 23 weeks (2015‐2016 season). The epidemic threshold with the exclusion of the pandemic season was 6.25%. It had a very high sensitivity of 85% and a high specificity of 69%. The different levels of intensity were established as follows: low, if ILI proportion is below 9.74%, medium below 12.05%; high below 13.27%; and very high above this last rate.

**Conclusions:**

This is the first mathematically based study of seasonal threshold of influenza in Tunisia. As in other studies in different countries, the model has shown both good specificity and sensitivity, which allows timely and accurate detection of the start of influenza seasons. The findings will contribute to the development of more efficient measures for influenza prevention and control.

## 1 INTRODUCTION

Seasonal influenza continues to be a public health problem worldwide. Although in most cases, it leads to an increased number of consultations, it may cause severe illness and death especially among high‐risk groups. In fact, the World Health Organization (WHO) has recently updated global estimates to more than 3 million severe cases and from 290 000 to 650 000 respiratory deaths due to influenza each year.^1^ These annual epidemics mobilize considerable resources from health services and even a small‐scale epidemic can have a significant socio‐economic burden.

Ongoing monitoring and assessment of seasonal influenza are therefore essential to ensure early warning of epidemics and tailored preventive and control measures in real‐time. The last 2009 pandemic revealed many deficiencies in most countries' influenza surveillance systems, especially the capacity to estimate the severity of the season in a timely manner. For this reason, the WHO has progressively developed a framework on pandemic influenza severity assessment (PISA) and recommended member states to apply the proposed tools and measures.^2^ The framework is based on different steps, including setting thresholds for selected parameters and applying them in the routine surveillance of seasonal epidemics.

Various mathematical and statistical models have been developed to establish thresholds for influenza activity and study the dynamics of the disease.^3^, ^4^, ^5^ This mathematical modeling provides valuable information and a strong support to the preparedness and response plan. Of the popular methods currently in use, the moving epidemic method (MEM) is one of the most recommended and so far had provided a robust signal to detect influenza epidemics in many countries.^6^, ^7^ First developed in Spain in 2001, the MEM was adopted by the same authors to determine influenza thresholds in many European countries.^8^ One of its strengths is its ability to also define different intensity levels in a given region or country and the possibility to compare them between countries and/or seasons.^9^, ^10^

In Tunisia, influenza surveillance was first based on the virological surveillance ensured by the National Influenza Centre (NIC) recognized by the WHO since 1980 and supported by the Primary Health Care Direction of Ministry of Health. Starting from the late 1990s, the epidemiological surveillance was established through the network of Influenza‐like illness (ILI) sentinel sites at primary healthcare centers in the 24 governorates of the country. This network was progressively improved mainly by reducing the number from 268 in 1999 to 113 ILI sites in 2014, better representativeness and training of all staff involved in the surveillance.^11^, ^12^ Each year, the proportion of ILI among the total number of consultations at ILI sentinel sites determines the intensity of influenza season. The epidemic threshold of 10% adopted since then was based on combination of criteria and a national approach.^13^, ^14^

Given the importance of seasonality and intensity levels in influenza severity assessment, our study aimed to determine the epidemic and intensity thresholds of influenza season in Tunisia by applying the moving epidemic method (MEM) based on ILI historical surveillance data of the last 9 years (2009‐2018).

## 2 METHODS

### 2.1 Available data

Influenza surveillance in Tunisia is carried out each year from 1st October (week 40) to 30th April (week 18) over a period of 30 weeks. Data collection is based on standardized forms of weekly aggregated data of ILI cases. These paper forms are sent from ILI sites at the local level to the regional directions in each governorate then to the Primary Health Care Direction at the national level. Aggregated data forms consist of general information including ILI site, governorate, the number of ILI cases and the total number of outpatients by gender and age groups (0‐5 years; 6‐16 years and ≥16 years). In Tunisia, case definition of ILI was an outpatient with fever (≥38°C) and cough or sore throat with onset less than 5 days prior to presentation in the absence of a specific diagnosis.^11^ Since 2014, the case definition recommended by WHO has been used instead: acute respiratory illness, and measured fever ≥38°C, and cough, and onset in previous 10 days.^12^ Collected data are analyzed to compute weekly ILI proportions compared with all outpatient acute consultations at both the national and regional levels. We analyzed data from up to nine influenza seasons (2009‐10 to 2017‐18).

### 2.2 Moving epidemic method

We applied the moving epidemic method (MEM) to establish epidemic and intensity thresholds, based on previous publications and the WHO's interim guidance for influenza severity assessment.^2^, ^8^, ^9^ For that, we used the R Language implementation of MEM (package “mem”) which is available online for free.^8^, ^15^ This method, based on a complex mathematical algorithm, can be summarized in three steps. First, determine the start, scope and end of the influenza epidemics by dividing the season in three periods (pre‐epidemic, epidemic and post‐epidemic periods). Then, epidemic thresholds are computed using the pre‐ and post‐epidemic values of historical seasons. Only a set of pre‐ and post‐epidemic values are used, of which we chose the highest n values for each season, with n = 30/number of seasons.^8^ This step requires moving the epidemic seasons in order to match the epidemic periods, after which we compute the geometric mean of weekly rates as well as different levels of confidence intervals (50%, 90% and 95%). Last, thresholds for the different intensity levels are determined by the upper limits of these confidence intervals; each upper limit represents the threshold of one level of intensity of the epidemic. Five levels of intensity are thus defined:

– Baseline: below the epidemic threshold
– Low level: between the epidemic and the medium thresholds
– Medium level: between the medium and the high thresholds
– High level: between the high and the very high thresholds
– Very high level: above the very high threshold

For the present work, we have described the epidemic and intensity thresholds from historical data of a period beginning in October 2009 and ending in April 2018.

The epidemic period is defined as the period of weeks with increased weekly values in a season. The periods of weeks before and after the epidemic period represent the pre‐epidemic and post‐epidemic periods, respectively. The epidemic threshold is the value which defines the start of the epidemic period while intensity thresholds represent the values marking the limit of the intensity levels. Besides, epidemic percentage is the sum of values in the epidemic period over the total sum of values of the whole influenza season, which reflects the coverage percentage of the epidemic period.

### 2.3 Cross‐validation procedure of the model

The goodness of the model was assessed using a cross validation procedure. This procedure is based on the extraction of each season from the historical series and using it as "a target season", for which we calculate the beginning and end of the epidemic period. Subsequently, the pre‐ and post‐epidemic thresholds are calculated on the basis of the remaining seasons and excluding the target season. These steps were repeated for all the available seasons.

Values of the target season inside and outside of the defined epidemic period were compared to the thresholds calculated using all historical information but the target season.

Aiming to evaluate the performance of the epidemic threshold to detect epidemics, we studied the sensitivity (Se), specificity (Sp), the positive predictive value (PPV) and the negative predictive value (NPV). The sensitivity consists of the model's ability to categorize epidemic weeks while the specificity is the model's ability to categorize non‐epidemic weeks. According to the cross‐validation analysis described by Vega et al,^8^ sensitivity is computed by dividing the number of epidemic weeks above the pre‐epidemic threshold and above the post‐epidemic threshold by the number of epidemic weeks. Specificity is the number of non‐epidemic weeks below the pre‐epidemic threshold and below the post‐epidemic threshold divided by the number of non‐epidemic weeks.

The number of weeks of the epidemic above the threshold, divided by the number of weeks above the threshold is the positive predictive value, expressing the proportion of epidemic weeks correctly classified by the model. On the other hand, the negative predictive value consists of the number of non‐epidemic weeks below the threshold, divided by the number of weeks below the threshold, corresponding to the proportion of non‐epidemic weeks correctly classified by the model.

To optimize the goodness of the model, we also looked at the optimum slope parameter to find the value that maximizes the sensitivity and specificity. It is an inner parameter ranging from 2% to 4%.^8^ Based on our data, it was 2.8% for both models with the inclusion and the exclusion of pandemic year 2009‐2010. The performance of the model was assessed by the Youden index (J = Sp + Se − 1), which reflects the variation of false positives and false negatives.

## 3 RESULTS

### 3.1 Descriptive analysis of the epidemic movement of influenza in Tunisia

The beginning, the end and the extent of the epidemic seasons differed from one season to another (Table 1).

**TABLE 1:** Characteristics of influenza epidemics in Tunis from 2009‐10 to 2017‐18 seasons

Season	Epidemic start	Epidemic end	Epidemic duration	Peak week	Peak^a^	Epidemic %^b^
2009‐2010	48	6	11	51	30.3%	74.77%
2010‐2011	46	15	22	9	9.2%	76.63%
2011‐2012	46	11	18	5	10.3%	77.52%
2012‐2013	49	15	19	8	15.1%	76.63%
2013‐2014	46	14	21	6	9.5%	76.62%
2014‐2015	45	14	22	8	10.3%	76.61%
2015‐2016	47	17	23	12	10.2%	76.52%
2016‐2017	44	10	19	3	10.9%	76.51%
2017‐2018	43	10	20	51	9.64%	76.62%

^a^ Proportion of ILI among the total number of consultations at ILI sentinel sites.

^b^ Coverage percentage of the epidemic period.

In most cases, influenza epidemic started around week 46. The first activity of influenza epidemic was observed in 2017‐2018 at week 43 and the latest one in 2012‐2013 at week 49.

Influenza epidemic during the pandemic year 2009‐2010 started at week 48 and ended at week 6. The duration of epidemic seasons ranged from 11 weeks (2009‐2010 season) to 23 weeks (2015‐2016 season). Epidemic percentage varied between 74.77% and 77.52%, corresponding to a high coverage of the epidemic period.

Almost all the seasons were one‐wave seasons except 2010‐2011 and 2014‐2015 with more than one wave. Most often, epidemic peaks were observed between week 3 and week 9. However, some seasons peaked earlier (week 51 in 2009‐2010 and 2017‐2018) and other seasons peaked later (week 12 in 2015‐16). The epidemic seasons seem to follow a pattern with a rapid increase at the beginning and a slowly decrease at the end of the season.

**FIGURE 1:**
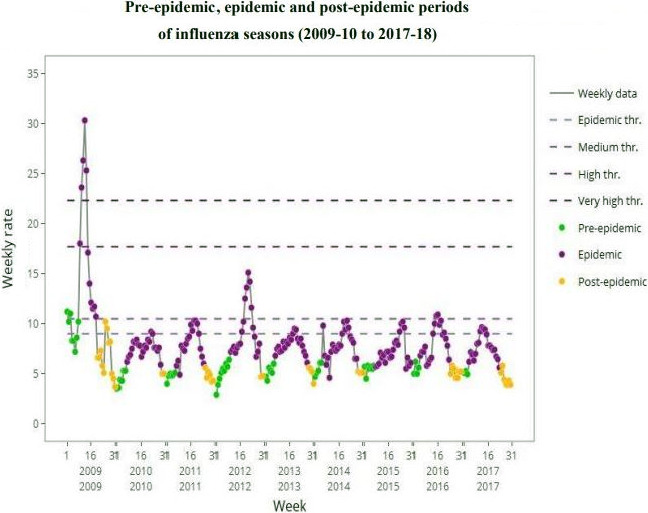
Pre‐epidemic, epidemic and post‐epidemic periods of influenza seasons (2009‐10 to 2017‐18)

Besides, Figure 1 displays the curves of all the studied seasons based on the accumulated maximum percentage rate method, specifying the pre‐ and post‐epidemic periods as well as the corresponding epidemic periods. ILI consultation rates differed also with the season, with the highest peak registered during 2009‐2010 (30.3%) and the lowest in 2010‐2011 season (9.2%).

Except the pandemic season which was considerate of a very high intensity, most of the seasons were described as low. This was useful to characterize the dynamics of influenza over time.

### 3.2 Epidemic and intensity thresholds

The different thresholds and intensity levels were determined using two models; one including and the other excluding the 2009‐10 pandemic season.

**FIGURE 2:**
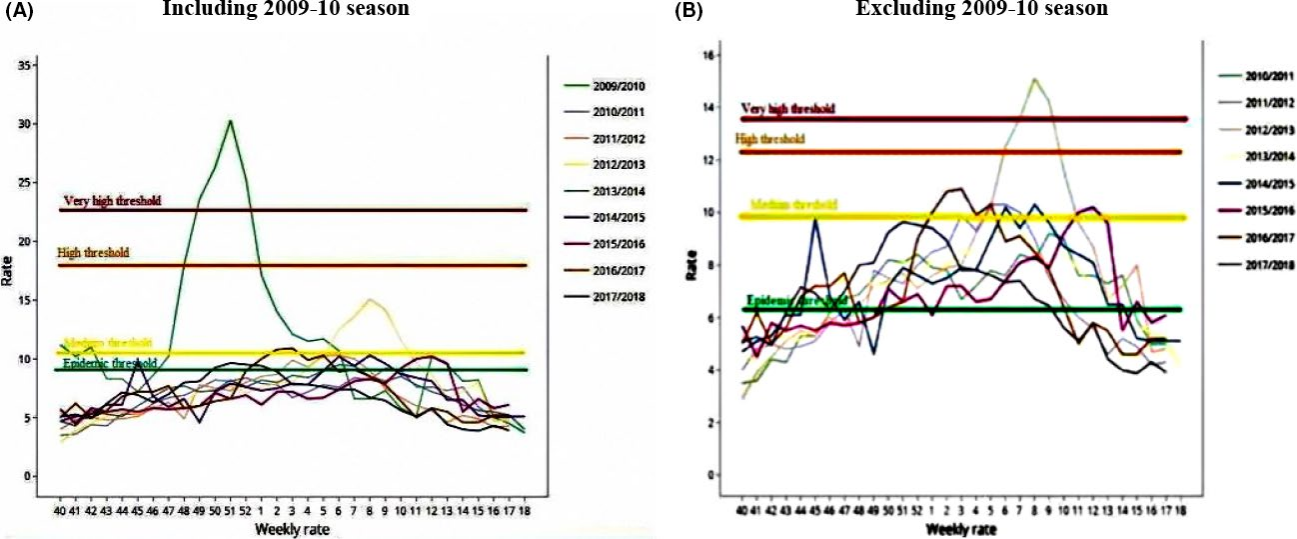
Epidemic movement including and excluding 2009‐2010 season. (A) Including 2009‐2010 season and (B) Excluding 2009‐2010 season

**FIGURE 3:**
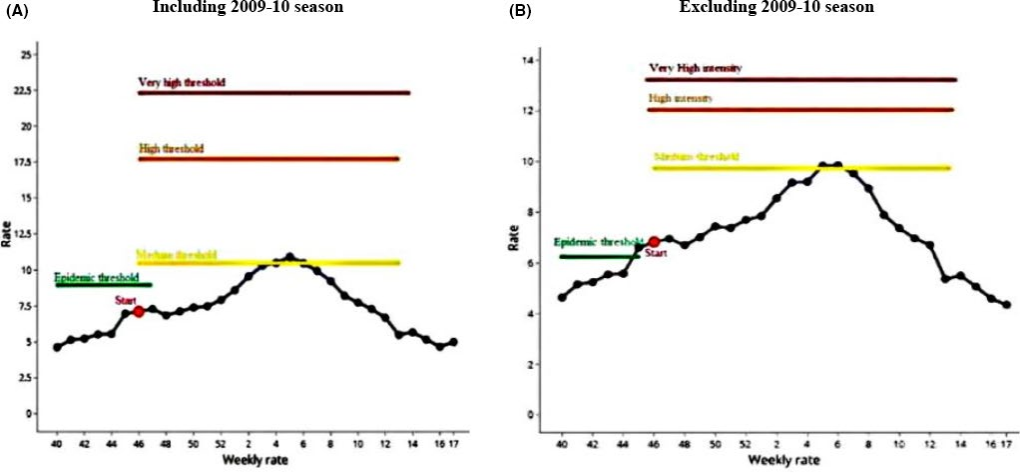
Average curve including and excluding 2009‐10 season. (A) Including 2009‐2010 season and (B) Excluding 2009‐2010 season

When including the pandemic season, the average duration of influenza epidemic during a typical season was 20 weeks (Figures 2A and 3A). This optimal duration of 20 weeks covers 76.61% of total sum of proportions.

As a result of this analysis, epidemic threshold was 8.99% if we include the pandemic season and post‐epidemic threshold was 8.25%.

Medium, high and very high intensity thresholds were 10.48%, 17.69%, and 22.3%, respectively (Table 2). Regarding the pandemic influenza season 2009‐2010, higher thresholds were observed comparing to the other seasons.

**TABLE 2:** Influenza epidemic thresholds and intensity levels including and excluding the pandemic year

	Epidemic threshold	Medium intensity threshold	High intensity threshold	Very high intensity threshold
Including 2009‐2010	8.99%	10.48%	17.69%	22.3%
Excluding 2009‐2010	6.25%	9.74%	12.05%	13.24%

When we excluded the pandemic season 2009‐2010, the average curve lasted 20 weeks and it covered 76.62% of the total rates (Figures 2B and 3B). The different parameters and indicators of epidemic threshold calculation decreased.

The levels of intensity, if we exclude 2009‐2010 season, were established as follows: low, if ILI proportion was below 9.74%, medium below 12.05%; high below 13.27%; and very high above this last proportion. These different intensity thresholds increased to 10.48% for the medium intensity level, to 17.69% for the high intensity level, to 22.3% for the very high threshold when including the pandemic season (Table 2).

### 3.3 Cross validation of the model

Table 3 presents the contribution of the different influenza seasons to calculate the epidemic threshold and how each season can affect this calculation. By proceeding to the comparison of the target season's rate during the epidemic and non‐epidemic period to the thresholds computed and excluding the target season, we observed differences in some seasons and others not. For instance, the pandemic season 2009‐2010 affected the estimate of the epidemic threshold. In fact, its exclusion lead to an important decrease of the epidemic threshold to 6.25% as well as different intensity thresholds.

**TABLE 3:** Contribution and influence of influenza seasons in the estimate of the influenza epidemic threshold: cross‐validation procedure

Season	Peak^a^ (%)	Peak week	Epidemic threshold	Medium threshold	High threshold	Very high threshold	Level	Description
2009/2010	30.3	51	6.25	9.74	12.05	13.24	5	Very high
2010/2011	9.2	9	9.14	10.62	17.91	22.59	2	Low
2011/2012	10.3	5	9.16	10.4	17.84	22.65	2	Low
2012/2013	15.1	8	9.1	10.02	16.99	21.46	3	Medium
2013/2014	9.5	6	9.14	10.55	17.91	22.63	2	Low
2014/2015	10.3	8	9.12	10.42	17.86	22.65	2	Low
2015/2016	10.2	12	9.13	10.45	17.88	22.66	2	Low
2016/2017	10.9	3	9.13	10.35	17.78	22.59	3	Medium
2017/2018	9.6	51	9.09	10.5	17.9	22.67	2	Low

^a^ Proportion of ILI among the total number of consultations at ILI sentinel sites.

This validation also allowed us to characterize the overall intensity of each season. Out of the nine seasons, six were low, two as medium, and one very high intensity (Table 3).

The MEM provided a sensitivity of 85% in detecting the epidemic period. This sensitivity during the overall seasons and for each influenza season increased from 39% to 85% if we exclude the pandemic year. However, the specificity was higher with inclusion of 2009‐10 and decreased from 87% to 69%. Simultaneously, there was a slight change in the VPP but the VPN significantly increased when excluding 2009‐10. Other indicators also increased if we did not consider the 2009‐10 season (Table 4).

**TABLE 4:** Goodness of the model

	Sensitivity	Specificity	Positive predictive value	Negative predictive value	Youden index
Including 2009‐2010	39%	87%	77%	52%	23%
Excluding 2009‐2010	85%	69%	79%	76%	53%

## 4 DISCUSSION

For almost two decades, the intensity of influenza activity in Tunisia has been estimated from data of the Sentinel Surveillance System based on a seasonal threshold of 10%, which was set up initially on a combination of criteria. The present study aimed to determine the epidemic threshold of influenza in Tunisia by the moving epidemic method using data of the nine past seasons (from 2009 to 2018).

This choice was largely motivated by the type of data available by the Tunisian influenza surveillance system. In fact, the basic requirements of the MEM are simple and reliable epidemiological data for a time period between 5 and 10 years, preferably ILI data.^8^

The MEM is a tool developed to better understand annual influenza epidemics and assess the epidemic status and intensity on a weekly basis.^8^, ^9^ The method was progressively improved and implemented in European documents by the ECDC and WHO.^15^ Later on, it became widely used in many countries outside Europe such as USA, Australia, New Zealand, and Canada.^6^, ^10^, ^16^, ^17^ Other countries have opted for the method proposed by WHO and based on the peak mean values of influenza activity.^5^, ^18^, ^19^

The determined epidemic threshold with the exclusion of the pandemic season was 6.25%. It showed a very high sensitivity (85%) and a high specificity (69%). However, when including 2009‐10, the threshold increased to 8.99% with a sensitivity and specificity of 39% and 87%, respectively. The different levels of intensity were also affected with a considerable increase. This is understandable since there was higher ILI rates registered during this year and thus higher pre‐ and post‐epidemic thresholds comparing to the other seasons. This confirms the importance of the pandemic year in the analysis and the need to exclude it when estimating the parameters and indicators of epidemic threshold.^5^, ^8^

The majority of authors used ILI consultations in primary healthcare settings expressed per 100 000 population ^8^, ^20^, ^21^, ^22^ or per 1000,^16^ which made the comparison with our results difficult, in addition to other differences in health systems, data collection methods as well as socio‐demographic characteristics, since most studies were from Europe or other developed countries. Studies on establishing influenza thresholds using the MEM in North Africa and the Eastern Mediterranean Region are limited. So far, only one study conducted in Egypt was available but aims and methodology were different.^23^ It would therefore be useful to establish one common method for ILI data analysis and interpretation in our region as was done in Europe.^9^, ^22^

Applying the MEM to define the thresholds also allowed us to visualize influenza activity in Tunisia for the past nine seasons. Overall, influenza seasons seem to be mostly one‐wave, homogenous with a seasonal pattern. Epidemics usually start between the 43rd and the 49th week and last from 11 to 23 weeks. The duration of epidemic seasons was comparable to the range reported in other studies (6‐25 weeks)^5^, ^7^, ^19^ and more specifically 12‐19 weeks in Europe.^24^ Except the pandemic season which was considerate of a very high intensity, most of the seasons were described as low. This was useful to characterize the dynamics of influenza over time. This seasonality and epidemiological patterns are a common thread in neighboring countries and most regions of the Northern Hemisphere sharing the same winter timing.^24^, ^25^, ^26^

The specificity of the determined epidemic threshold was lower than its sensitivity. Sensitivity is important for detecting epidemics but specificity is crucial to avoid false alerts. In fact, once an epidemic is declared, the media's interest increases and prevention and control measures are implemented, especially vaccination campaigns and antiviral use.^8^ That is why it is important to avoid false alerts and to use these attributes wisely trying to find the good balance between specificity and sensitivity.

Besides, it is important to underline that the specificity of the model is related to the case definition used. The lower is the specificity of the case definition, the lower is the specificity of the model. Although a new case definition was used since 2014, the changes enhanced sensitivity without greatly compromising the specificity.^27^ We therefore consider that the specificity of the model did not change over time. The specificity found in our results can be explained by these rather sensitive case definitions, which may increase the identification of other respiratory pathogens especially the respiratory syncytial virus (RSV) and lead to more consultations of acute respiratory infections (ARI) than ILI. Other factors may affect the outpatient rates including public anxiety and excessive awareness and sensitization of physicians in case of false alerts. The continuous training of ILI sites especially on the precise definitions improves the MEM's performance and precision in the epidemic threshold's estimation.

This low specificity may represent the main limitation of our study. Most authors concluded to models with very high specificity. In Vega's study about establishing thresholds in 19 European countries, the lowest specificity was 89.6% in Kazakhstan with an overall specificity of 95.5%.^8^ Other limitations may have resulted of changes in demographics, case reporting and especially ILI case definition, which was modified in 2014, in the second half of the study period.

The circulation of a new influenza virus, as it was observed during the 2009‐2010 pandemic in many countries, may also generate abnormal epidemiological data and falsely positive results.

In these situations, additional virological data are necessary to confirm the start of the epidemic period, especially that our results showed a better performance of the MEM model excluding the pandemic season than the one including this season.

## 5 CONCLUSION

In summary, the moving epidemic method is a simple method offering a flexible procedure to calculate epidemic thresholds based on historical epidemiological data. Its strength lies in its ability to also determine different intensity thresholds useful to the weekly monitoring of the season's intensity.

Our study is the first mathematically based study of seasonal threshold of influenza in Tunisia using historical ILI weekly data. The determined epidemic threshold was 6.25%, differing from the threshold of 10% adopted until now. The high sensitivity and specificity of this threshold in the detection of epidemics make it robust and reliable.

Indicating the start and assessing the intensity of influenza seasons remain a high priority for Ministries of Health, not only at the national level for timely preventive and control measures but also at the international level by contributing to the pandemic preparedness. The next step is therefore to implement the use of the determined epidemic threshold for public health purposes with monitoring the next seasons.
